# Real-World Comparative Efficacy and Safety of Upadacitinib, Tofacitinib, and Filgotinib in Patients With Ulcerative Colitis

**DOI:** 10.7759/cureus.102069

**Published:** 2026-01-22

**Authors:** Fraz Ahmad, Tausif Hussain, Chamith H Gunaratne, Joseph Collum, Abdul Ghaffar, Khaled Gharbia

**Affiliations:** 1 Gastroenterology and Hepatology, Royal Blackburn Hospital, Blackburn, GBR; 2 Internal Medicine, East Lancashire Hospitals NHS Trust, Blackburn, GBR; 3 Gastroenterology and Hepatology, East Lancashire Hospitals NHS Trust, Blackburn, GBR

**Keywords:** gastrointestinal, inflammatory bowel disease, janus kinase inhibitors, real-world study, tofacitinib, ulcerative colitis, upadacitinib

## Abstract

Janus kinase (JAK) inhibitors, including upadacitinib, tofacitinib, and filgotinib, represent an emerging class of effective oral therapies for the treatment of moderate to severe ulcerative colitis (UC). We report a real-world retrospective study evaluating the comparative efficacy and safety of these three JAK inhibitors in 52 patients ranging from mild to severe UC. Significant improvements in inflammatory biomarkers were observed across all treatment groups, with biochemical response (reduction in faecal calprotectin (FCP)) achieved in 21/22 upadacitinib-treated patients, 22/24 tofacitinib-treated patients, and 5/6 filgotinib-treated patients. High-grade biochemical response (≥75% FCP reduction or normalization) was most frequently observed with upadacitinib (86.4%; N=19), followed by tofacitinib (62.5%; N=15) and filgotinib (50%; N=3). Endoscopic reassessment was available in a subset of patients, and the majority showed improvement or resolution of endoscopic inflammation, particularly in the upadacitinib and tofacitinib groups. The safety profile was consistent with recognized JAK inhibitor side effects, with hyperlipidemia being the most common adverse event and intermittent cytopenias noted in some patients; no serious opportunistic infections or thromboembolic events were recorded. These findings suggest that all three JAK inhibitors are effective therapeutic options in real-world practice, with upadacitinib demonstrating the strongest overall biochemical response among this treatment-experienced UC cohort. Larger prospective studies are warranted to confirm the long-term efficacy of these agents.

## Introduction

Chronic idiopathic inflammatory bowel disease (IBD) is a clinically challenging and heterogeneous immune-mediated condition with two distinct variants: Crohn's disease and ulcerative colitis (UC). Maintenance immunosuppressive therapy is the cornerstone of long-term management for the majority of IBD patients. The goals of such therapy include the reduction of disease flare-ups and steroid exposure and prevention of the long-term sequelae of inflammatory intestinal damage. The options for medical therapy have expanded significantly in recent years, driven by the expansion of classical biologic therapies and the emergence of so-called small-molecule drugs [1,2].

Janus kinase (JAK) inhibitors are the most important new therapeutic group and are distinguished by their novel mechanism of action and oral route of delivery. The JAK inhibitors also differ from traditional biologics in their rapid onset of action and greatly decreased risk of immunogenicity.

The therapeutic target of such drugs is JAKs, non-receptor tyrosine kinases that mediate cytokine signaling via the JAK/signal transducer and activator of transcription (STAT) pathway and ultimately control multiple transcriptional processes [3]. Monoclonal antibodies remain a crucial component of IBD treatment; however, up to 40% of individuals experience primary non-response to anti-tumor necrosis factor (TNF) therapy, and even higher proportions subsequently develop loss of response [4].

Upadacitinib is a selective and reversible inhibitor of the Janus-associated tyrosine kinase JAK1. Its use was licensed by the National Institute for Health and Care Excellence (NICE) in January 2023. In terms of dosing, upadacitinib is started at an induction dose of 45 mg daily for eight weeks and either 15 mg or 30 mg daily for maintenance. If an adequate therapeutic response is not achieved after eight weeks, the initial dose can be extended for an additional eight weeks [5].

Tofacitinib selectively inhibits the Janus-associated tyrosine kinases JAK1 and JAK3. Its use was licensed by NICE in November 2018. In terms of dosing, tofacitinib is started with an induction of 10 mg twice daily for eight weeks and then a maintenance dose of 5 mg twice daily. If an adequate therapeutic response is not achieved after eight weeks, the initial dose can be extended for an additional eight weeks [6].

Filgotinib is a selective inhibitor of the Janus-associated tyrosine kinase JAK1. Its use was licensed for the treatment of UC by NICE in June 2022. In terms of dosing, filgotinib is started at 200 mg once daily for 10 weeks. If an adequate therapeutic response is not achieved after 10 weeks, the initial dose can be extended for an additional 12 weeks [7].

According to the 2025 British Society of Gastroenterology guidelines, upadacitinib is recommended for the induction and maintenance of remission in moderate to severe UC, supported by high-certainty evidence and a large magnitude of effect. In comparison, tofacitinib is suggested for induction and maintenance therapy based on moderate-certainty evidence demonstrating a large treatment effect. Filgotinib is also suggested for the induction and maintenance of remission, although the supporting evidence is of low certainty with a moderate clinical effect [8].

The primary aim of this study was to compare the efficacy and safety profile of the above three JAK inhibitors in the treatment of UC. This was assessed by measuring the changes in inflammatory biomarkers, improvement in clinical symptoms, and changes in endoscopic findings. The secondary aim was to evaluate the safety profile of these three JAK inhibitors by documenting the incidence and nature of any adverse events observed during treatment.

## Materials and methods

This was a retrospective, observational, single-center study conducted at the Royal Blackburn Hospital, East Lancashire Hospitals NHS Trust, Blackburn, United Kingdom. The study evaluated the real-world effectiveness and safety of upadacitinib, tofacitinib, and filgotinib in the treatment of UC. All data were obtained from routine clinical records, including electronic patient notes, laboratory systems, and endoscopy reporting databases.

Inclusion criteria

Patients were eligible for inclusion if they had a confirmed diagnosis of UC based on standard clinical, endoscopic, and histological criteria and had commenced treatment with one of the three licensed JAK inhibitors, upadacitinib, tofacitinib, or filgotinib, during the period of July 2019-October 2025. Patients were required to have baseline faecal calprotectin (FCP) and baseline endoscopic evaluation.

Exclusion criteria

Individuals with non-UC diagnoses, such as Crohn's disease or indeterminate colitis, and those with incomplete records lacking baseline data were excluded. All prescribing decisions, monitoring intervals, and follow-up arrangements were determined by the treating gastroenterology team in accordance with routine clinical care.

Outcomes

The primary outcome was biochemical response, assessed by comparing FCP values before and after the initiation of JAK inhibitor therapy. FCP was selected as the main biochemical marker due to its reliability, non-invasiveness, and strong correlation with mucosal inflammation in UC. Secondary outcomes included endoscopic response, assessed through follow-up colonoscopy when clinically indicated, and clinical response as documented in routine consultations and recorded symptom assessments.

Safety outcomes included the assessment of all documented adverse events, metabolic effects, cytopenias, and treatment intolerance, recorded in routine follow-up visits.

Analysis

FCP levels pre- and post-treatment were illustrated as continuous variables using bar charts. Endoscopic outcomes and adverse events were summarized descriptively as counts and percentages. No formal statistical testing was performed due to the retrospective nature of the study and variation in the availability of endoscopic reassessment. This study was undertaken as part of a clinical service evaluation, using data collected during routine clinical care, and is covered by the guidance of the NHS Health Research Authority [9].

## Results

**Figure 1:**
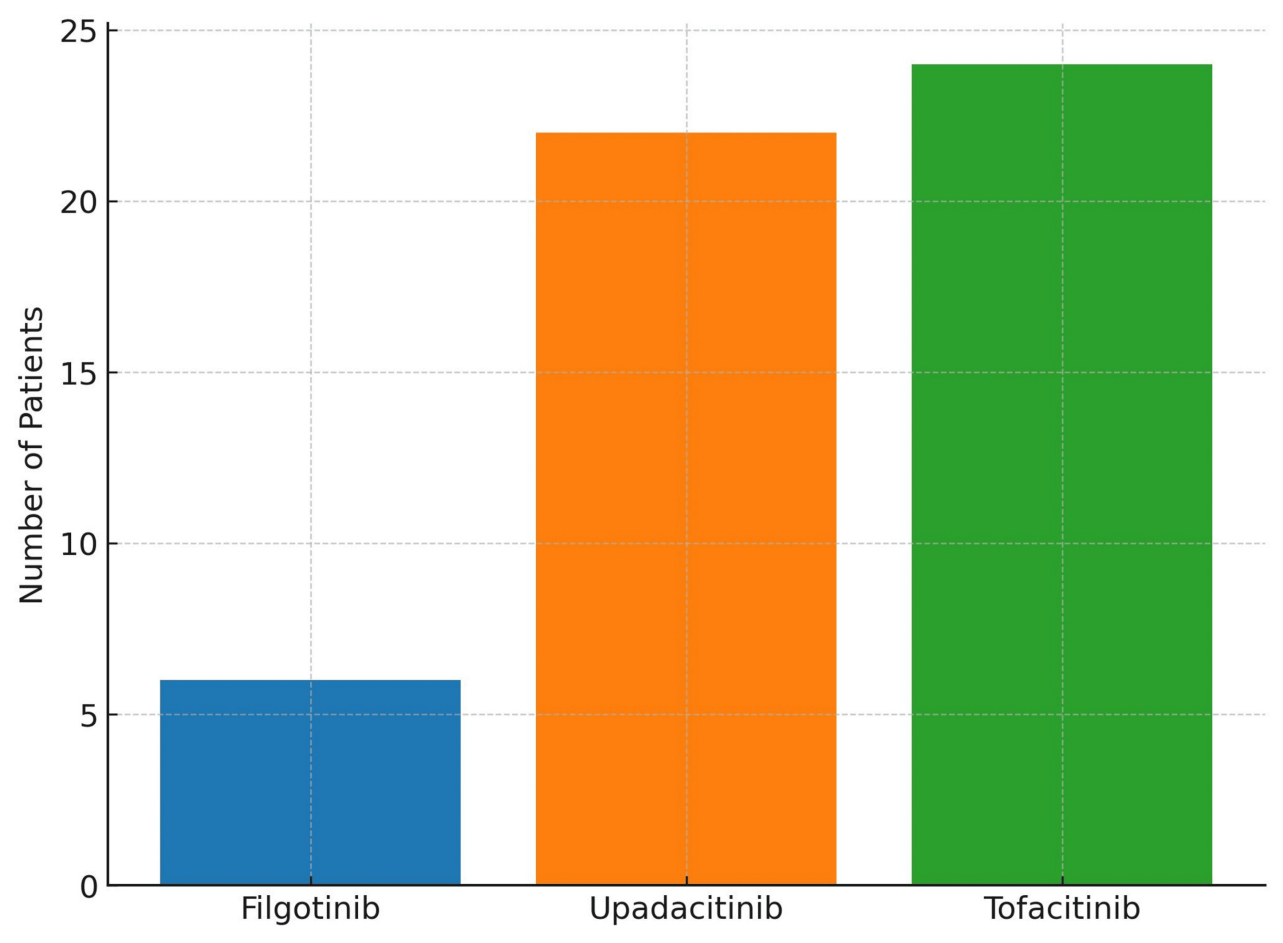
Patients receiving JAK inhibitor therapy (N=52) JAK: Janus kinase

**Figure 2:**
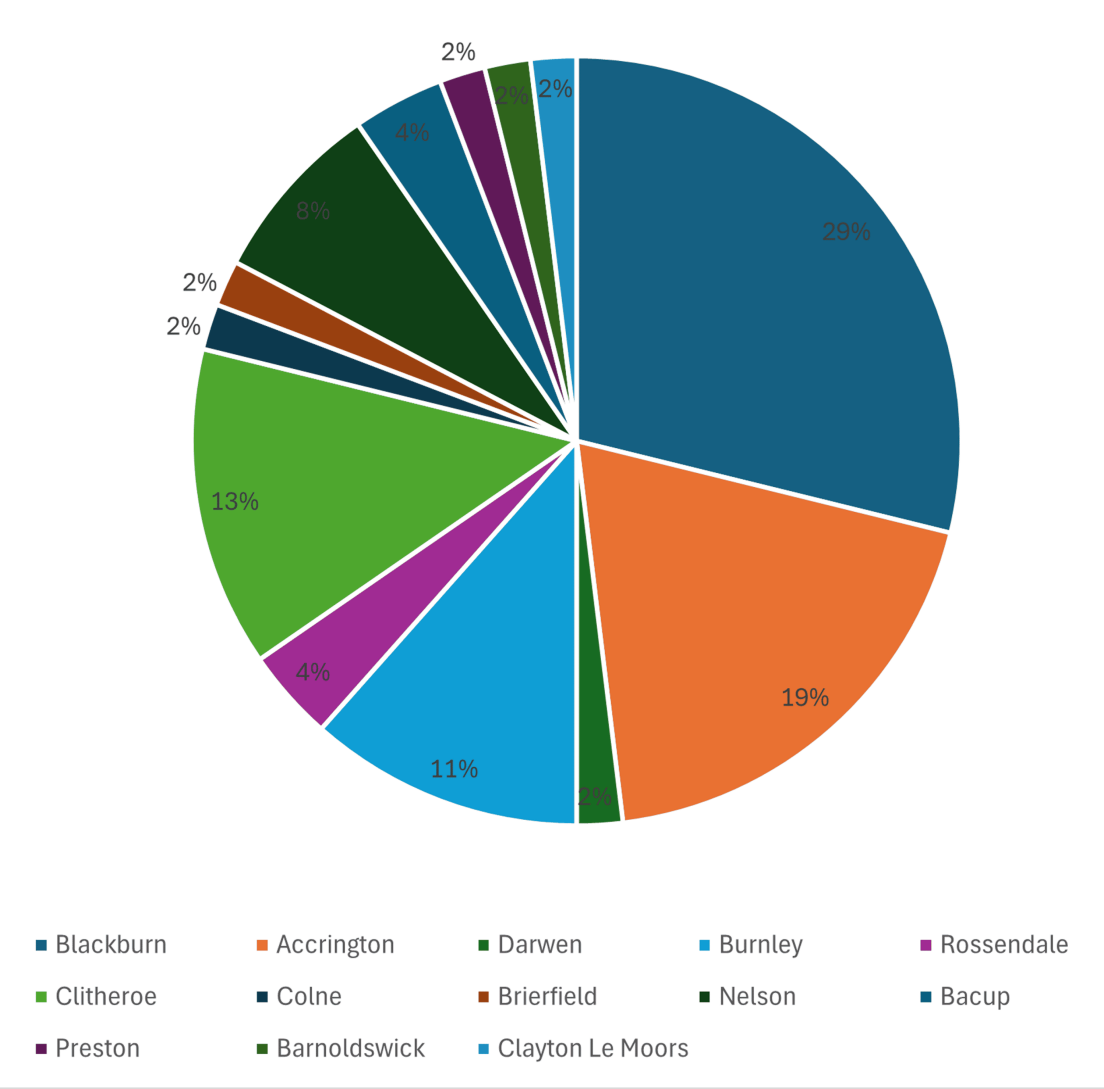
Demographic distribution of patients (N=52)

A total of 52 patients with UC receiving JAK inhibitor therapy were included in this study. Patients were predominantly 60% male (n=31) and 40% female (n=21) (Table 1). Of the 52 patients included in the analysis, six were treated with filgotinib, 22 with upadacitinib, and 24 with tofacitinib (Figure 1). Demographic mapping (Figure 2) reflected that the majority of patients were from Blackburn, Accrington, and Burnley.

**Table 1:** Baseline characteristics of the study cohort (total N=52) FCP: faecal calprotectin

Characteristic		Value
Total patients		52
Gender	Male	31 (60%)
	Female	21 (40%)
Cohort size	Tofacitinib	24 (46%)
	Upadacitinib	22 (42%)
	Filgotinib	6 (12%)
Prior exposure to advanced/biologic therapy	Yes	41 (78.8%)
	No	11 (21.2%)
≥2 prior advanced/biologic agents	Yes	27 (51.9%)
	No	25 (48.1%)
Locality	Blackburn	15 (29%)
	Accrington	10 (19%)
	Clitheroe	7 (13%)
	Burnley	6 (11%)
	Nelson	4 (8%)
FCP baseline availability		52 (100%)

**Figure 3:**
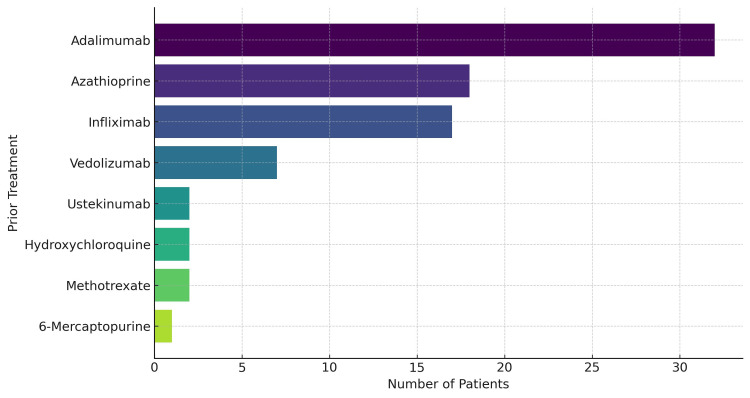
Frequency of biologic and immunomodulator treatments prior to being started on JAK inhibitors (N=41) JAK: Janus kinase

An analysis of prior therapies among the 52 patients receiving JAK inhibitor treatment demonstrated that around 41 patients (78.8%) had substantial previous exposure to advanced and biologic therapies (Figure 3). The most frequently used agents were adalimumab, azathioprine, and infliximab, indicating that the majority of patients had received at least one anti-TNF or immunomodulatory therapy before commencing JAK inhibitors. Additional prior biologics included vedolizumab and ustekinumab and conventional treatments like hydroxychloroquine, methotrexate, and 6-mercaptopurine. Collectively, these findings highlight that this cohort represented a treatment-experienced and partially biologic-refractory population, with many individuals having cycled through multiple therapeutic classes prior to starting JAK inhibitor therapy. Notably, 27 of these 41 patients (65.9%) had exposure to two or more advanced/biologic agents prior to starting JAK inhibitor therapy.

**Figure 4:**
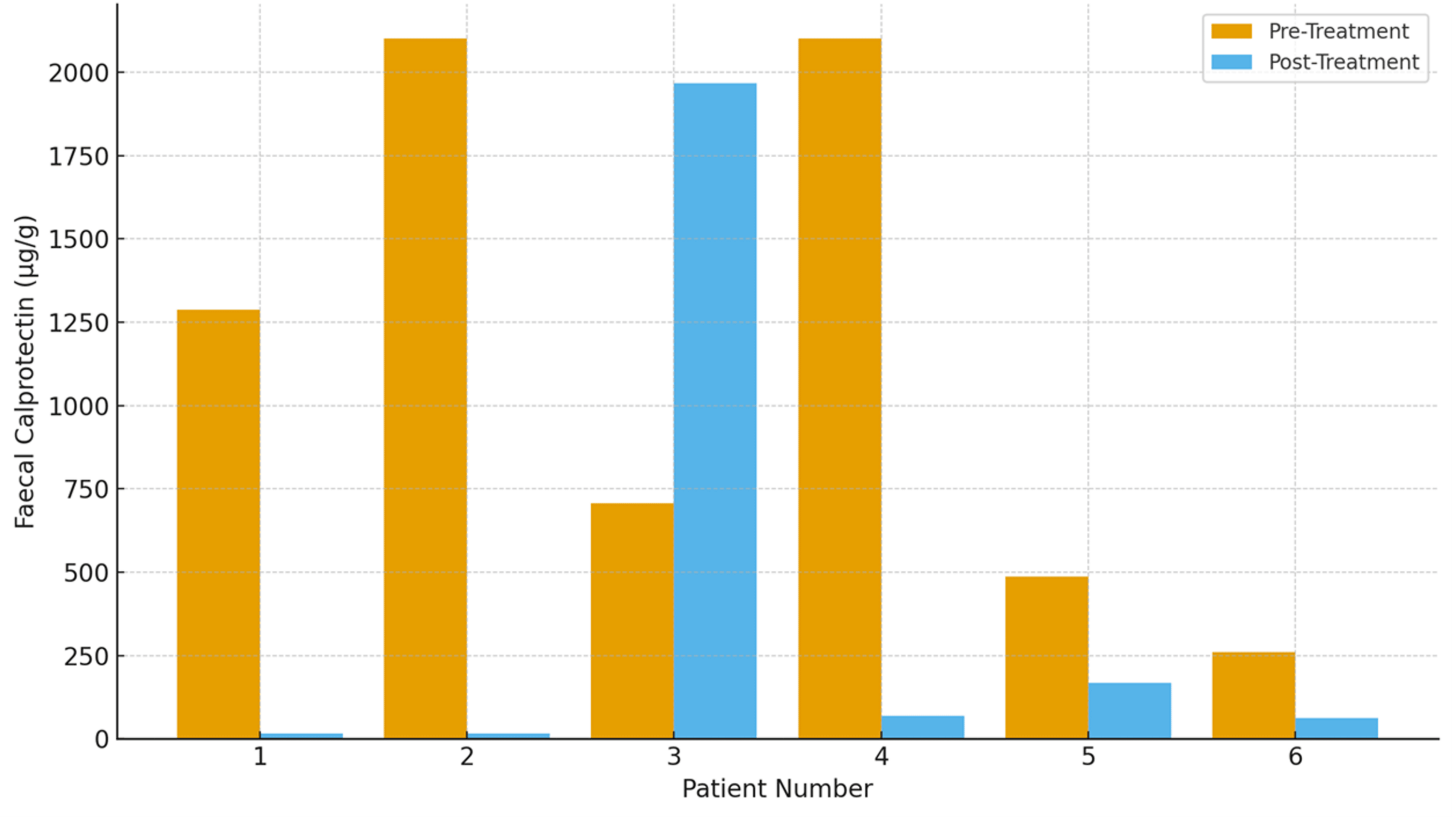
Faecal calprotectin levels before and after treatment with filgotinib (N=6)

Among the six patients treated with filgotinib, five patients (83.3%) demonstrated a reduction in FCP at follow-up (Figure 4). Of these, three patients (50%) achieved a high-grade biochemical response, defined as either the complete normalization of FCP or a ≥75% reduction from baseline. In contrast, one patient (16.7%) experienced worsening FCP levels, with persistent active inflammation confirmed on colonoscopy, and was subsequently switched to an alternative therapy for UC. A total of two patients (33.3%) underwent repeat endoscopic evaluation, of whom one patient (16.7%) demonstrated clear endoscopic improvement. Treatment-related adverse events included leucopenia in one patient (16.7%) and hypercholesterolemia in two patients (33.3%). Overall, filgotinib was associated with meaningful FCP improvement in the majority of treated patients, with a favorable tolerability profile in this small cohort.

**Figure 5:**
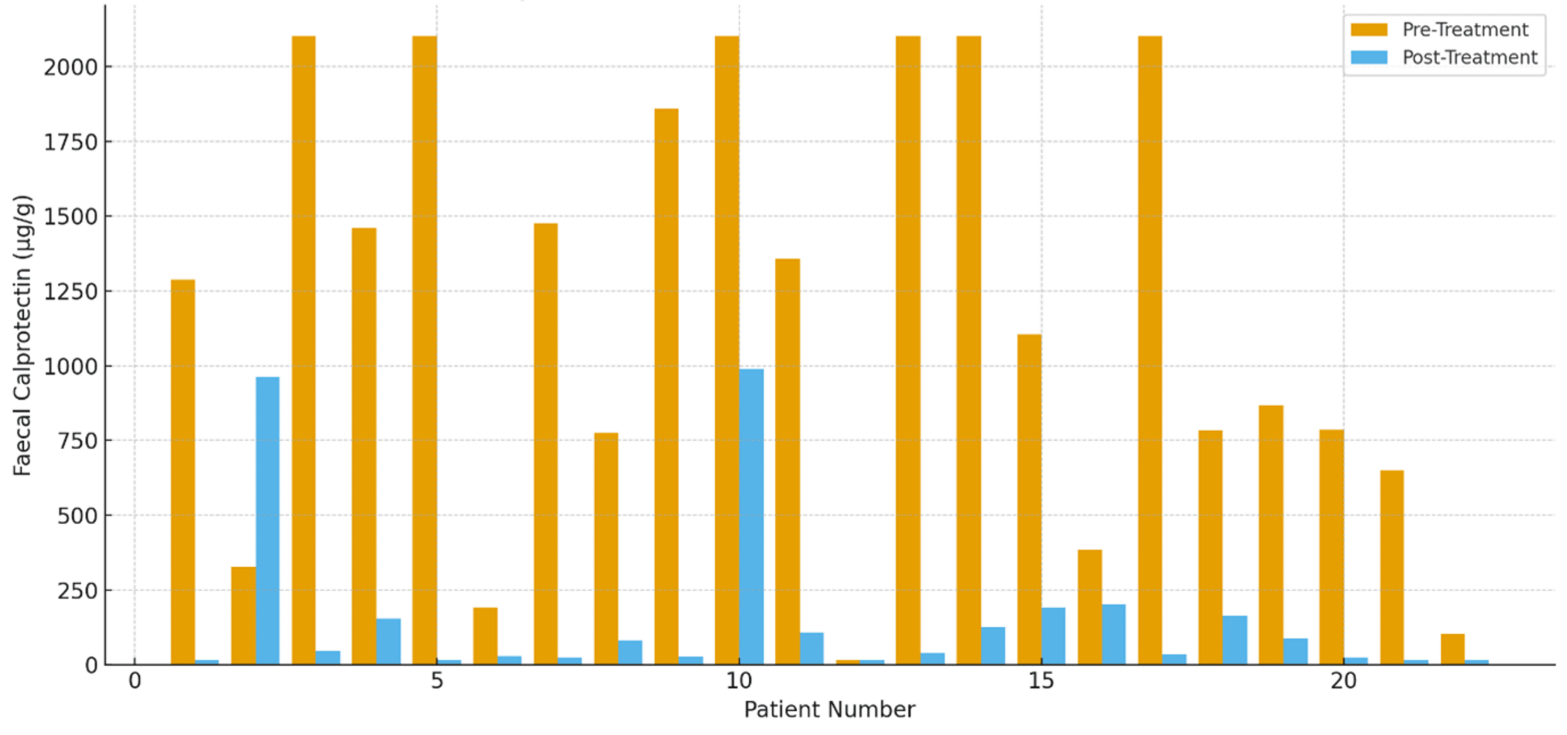
Faecal calprotectin levels before and after treatment with upadacitinib (N=22)

Among the 22 patients treated with upadacitinib, 21 patients (95.5%) demonstrated an improvement in FCP levels at follow-up, indicating a clinically meaningful response (Figure 5). A high-grade biochemical response was achieved in 19 patients (86.4%). Only one patient (4.5%) showed worsening in FCP levels. Four patients (18.2%) underwent repeat colonoscopic assessment, of whom three demonstrated no endoscopic evidence of active disease, consistent with mucosal quiescence. In terms of safety profile, 10 patients (45.5%) developed derangements in lipid profile, and five patients (22.7%) experienced intermittent cytopenias, which were monitored accordingly. Overall, these findings emphasize the strong real-world therapeutic effectiveness of upadacitinib.

**Figure 6:**
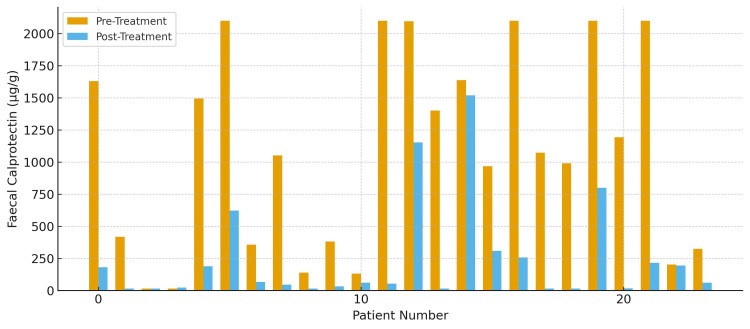
Faecal calprotectin levels before and after treatment with tofacitinib (N=24)

Among the 24 patients treated with tofacitinib, 22 patients (91.7%) demonstrated an improvement in FCP levels, while two patients (8.3%) showed no considerable improvement (Figure 6). A total of 15 patients (62.5%) achieved FCP normalization or a ≥75% reduction from baseline. Twelve patients (50%) underwent repeat colonoscopic evaluation, all of whom demonstrated improvement in endoscopic disease activity. Regarding safety, 15 patients (62.5%) developed derangements in lipid profile, and nine patients (37.5%) experienced intermittent cytopenias. Collectively, these findings indicate that the majority of UC patients experienced meaningful improvement with tofacitinib in real-world practice.

**Figure 7:**
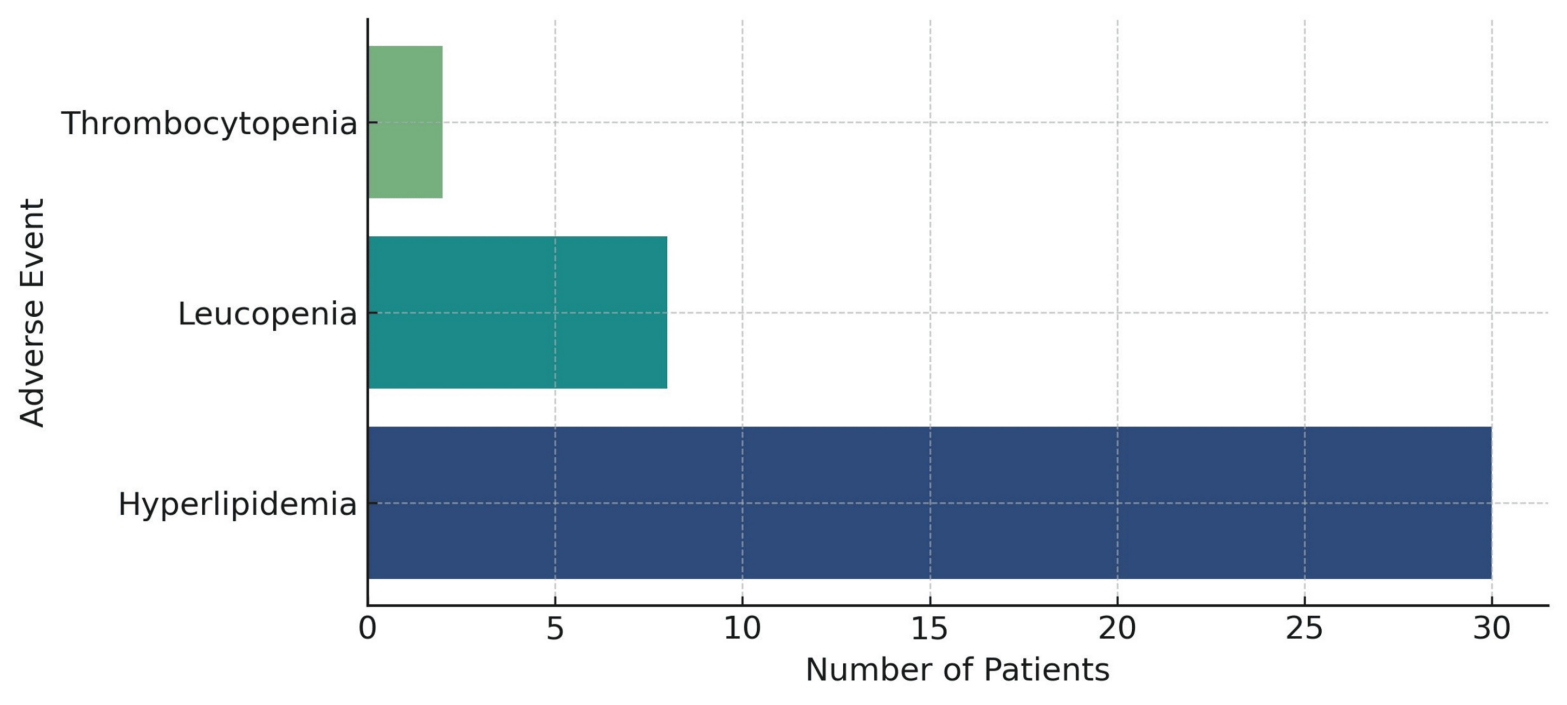
Frequency of adverse events among the patients (N=30)

In terms of side effects, hyperlipidemia was the most frequently reported adverse event, occurring in 30 of 52 patients (57.7%). Leucopenia was documented in eight patients (15.4%), while thrombocytopenia was observed in two patients (3.8%). Notably, 17 patients (32.7%) had no recorded side effects. Overall, the safety profile observed in this real-world cohort is consistent with the recognized side effects of JAK inhibitors, with dyslipidemia representing the predominant metabolic abnormality and intermittent cytopenias constituting the principal hematological abnormality (Figure 7).

## Discussion

This retrospective single-center study provides real-world comparative data on the three currently licensed JAK inhibitors for UC, upadacitinib, tofacitinib, and filgotinib, in a cohort of patients with moderate to severe UC, the majority of whom had previously failed several advanced therapies. All patients included in the study had received the FDA/NICE-approved induction doses of these JAK inhibitors.

Our primary outcome measure was the change in FCP, chosen for its sensitivity to colonic mucosal inflammation and practicality in routine clinical care. In our cohort, upadacitinib demonstrated the highest overall efficacy among the three JAK inhibitors, with 95.5% (N=21) of patients achieving a reduction in FCP and 86.4% (N=19) reaching a high-grade biochemical response. Tofacitinib also showed strong effectiveness, with 91.7% (N=22) of patients experiencing improvement in FCP and 62.5% (N=15) achieving a high-grade biochemical response, alongside universal endoscopic improvement in those who underwent follow-up colonoscopy. Filgotinib, although used in a smaller subset of patients, produced meaningful clinical benefit, with 83.3% (N=5) of patients showing biochemical improvement and 50% (N=3) achieving a high-grade response. When these results are considered collectively, upadacitinib appears to offer the greatest likelihood of achieving substantial biochemical remission in a real-world, treatment-experienced UC cohort. However, each of the three JAK inhibitors demonstrated clinically relevant improvement.

Among the patients who underwent repeat colonoscopy, most patients demonstrated improvement or resolution of endoscopic inflammation, especially in the upadacitinib and tofacitinib groups. However, not all patients underwent repeat colonoscopy, which reflects real-world pragmatism; once symptoms and biomarkers improve, clinicians and patients are often less inclined to pursue invasive reassessment.

It is notable that in our cohort, there was no specific washout period set for patients on previous biologic treatments, before starting JAK inhibitors. In daily clinical practice, clinicians rarely impose lengthy washout intervals between biologic agents, particularly when patients require prompt therapeutic escalation. The good response rates observed in this context suggest that prolonged washout intervals may not be essential. All biologic-naïve patients in our cohort (N=10) demonstrated improvement in FCP after initiating JAK inhibitor treatment, further supporting the utility of JAK inhibitors across treatment-experienced and treatment-naïve populations.

The outcomes of our study align well with emerging real-world and clinical data supporting the efficacy of JAK inhibitors in moderate to severe UC. A recent study conducted within the same NHS Trust evaluated outcomes with upadacitinib alone and concluded that it was an effective and well-tolerated option in patients who had failed multiple prior therapies, with biochemical and symptomatic improvement in the majority of cases; this patient cohort was incorporated within the present study [10].

In a recent multi-center retrospective cohort including 228 patients on upadacitinib, 215 on filgotinib, and 159 on tofacitinib, clinical remission at follow-up was highest in the upadacitinib group (72.8%), compared with 50.6% for filgotinib and 45.8% for tofacitinib, with upadacitinib showing higher efficacy and lower discontinuation rates than the other JAK inhibitors [11]. Similarly, in another study comparing filgotinib and upadacitinib, at eight-week follow-up, clinical remission was 65.7% for the upadacitinib group and 46.9% for the filgotinib group, respectively [12]. These findings are consistent with a recent systematic review and network meta-analysis involving over 14,000 patients, which ranked upadacitinib as the most effective therapy for inducing clinical remission among currently available biologics and small molecules [13]. These findings are similar to our results in which upadacitinib achieved the highest rate of high-grade biochemical response (86.4%; N=19).

Additionally, in another large Bayesian network meta-analysis comparing multiple biologics and small molecules, upadacitinib was ranked as the most efficacious regimen for achieving and sustaining clinical response and remission in both biologic-naïve and biologic-experienced patients. Given the more predictable pharmacokinetics of JAK inhibitors, it was hypothesized that patients receiving these may have faster resolution of symptoms compared with other treatment options [14]. Further comparative data from a large multi-center real-world cohort of 290 UC patients (tofacitinib N=150; filgotinib N=79; upadacitinib N=61) showed that upadacitinib was associated with the highest rates of clinical effectiveness at both short- and long-term follow-up. Notably, the authors highlighted the large use of off-label maintenance doses in current practice and proposed that it needs further safety investigations [15].

Furthermore, an updated network meta-analysis examining advanced therapies in UC found that JAK inhibitors, as a class, were generally superior to other advanced treatments for both the induction and maintenance of remission, in line with our observation that all three JAK inhibitors produced clinically meaningful benefit in this real-world cohort [16].

Hyperlipidemia represented the most common adverse event in our cohort (57.7%; N=30), followed by leucopenia (15.4%; N=8) and thrombocytopenia (3.8%; N=2), whereas nearly one-third of patients (N=17) experienced no adverse events. This pattern mirrors the established safety profile of JAK inhibitors, where metabolic derangements and mild cytopenias are frequently observed. Other important adverse events reported with JAK inhibitors include opportunistic infections, particularly herpes zoster, and cardiovascular and venous thromboembolic (VTE) events [17]. Overall, JAK inhibitors should be used following careful cardiovascular and thrombotic risk evaluation, with particular attention to older patients and those with pre-existing risk factors, in addition to ensuring vaccination against herpes zoster and regular laboratory monitoring.

Our experience with JAK inhibitors has been significantly positive, with the majority of patients achieving effective disease control. They also demonstrate a favorable and reasonably effective safety profile in our cohort, supporting their usefulness in managing UC flares and reducing the reliance on corticosteroids. When interpreted alongside existing real-world and clinical trial data, our findings reinforce the place of JAK inhibitors as highly effective oral treatment options for moderate to severe UC, particularly in patients who are refractory to conventional or biologic therapies.

Strengths and limitations

The primary strength of this study is the comparison of the efficacy of all three currently licensed JAK inhibitors (upadacitinib, tofacitinib, and filgotinib), within a single NHS center, using objective biochemical markers such as FCP and incorporating endoscopic outcomes where available. Another strength is the inclusion of patients with extensive prior exposure to biologic and advanced therapies, allowing the assessment of JAK inhibitor performance in a difficult-to-treat patient population. The use of standardized, guideline-directed dosing for all three agents also strengthens the outcome for this study.

However, several limitations must be acknowledged. The main limitation of this study was that it was a retrospective analysis in a single center, with a relatively small sample size. Additionally, endoscopic evaluation was not uniformly available for all the patients. Despite these limitations, the results from this study offer valuable real-world insight into the practical use of upadacitinib, tofacitinib, and filgotinib, in challenging UC patients who have not responded to prior treatments.

## Conclusions

In this retrospective study, JAK inhibitors (upadacitinib, tofacitinib, and filgotinib) were found to be effective and well-tolerated in patients with UC who had previously not responded to other advanced biologic therapies. Most patients experienced significant reductions in inflammatory biomarkers (FCP) and improvement in clinical symptoms. Upadacitinib demonstrated the highest rate of high-grade biochemical response, while tofacitinib and filgotinib also produced substantial reductions in FCP and favorable endoscopic outcomes in those reassessed. The overall safety profile was consistent with recognized side effects, with hyperlipidemia and mild cytopenias being the most commonly observed adverse events. There were no serious adverse events encountered in this cohort of patients.

These findings support the growing evidence that JAK inhibitors are effective and practical oral therapeutic options for moderate to severe UC patients, particularly in patients who have failed multiple biologics. Further prospective studies with larger cohorts and long-term follow-up would be beneficial to understand the outcomes and long-term effects of treatment with JAK inhibitors.
